# Modern Gynecology

**Published:** 1894-02

**Authors:** 


### Book Notices.

MODERN GYNECOLOGY. A TREATISE ON DISEASES OF WOMEN. Comprising the results of the latest investigations and treatment in this branch of Medical Science. By Charles H. Bushong, M. D., assistant gynecologist to the Demitt Dispensary, New York, formerly attending physician to the Northern Dispensary, and assistant to the Vanderbilt Clinic College of Physicians and Surgeons, New York. In one volume of 380 pages. 105 illustrations. Price, cloth $2.75. E. B. Treat, Publisher, 5 Cooper Union, New York.

The works on gynecology are now so numerous, and the field seems to have been so well covered by men of ability and learning, that the necessity, or even the room, for another book on this special line does not at first thought seem apparent. But, in presenting this book to the consideration of the medical profession of this country, Dr. Bushong gives some special and strong reasons why a work such as his is not only justifiable, but actually demanded. He points out the fact that many general practitioners, and especially those who were educated five to twenty years ago, when the facilities for teaching gynecology in our medical institutions were far inferior to what they are at this time, are deficient in a knowledge of diseases peculiar to women; that the average work on this subject is so voluminous that they have not sufficient time to read and study them, and thus secure the necessary information; hence a book short and concise, and at the same time giving all the latest and most important facts connected with this subject is a necessity.

A very large per cent. of the women prefer consulting their family physician to any one else, and many of them will refuse to be treated by another physician; then it is all important that this family physician should be prepared to give these cases the proper treatment, or at least be able to make out a diagnosis and give the necessary advice in regard to securing proper treatment. Many of these cases perfectly curable in the beginning, are, from neglect or incompetency, allowed to go on until they are past being cured, even in the most skillful hands.

Dr. Bushong's book is well printed and well written throughout. It is admirably adapted to the purpose for which it is intended, and we believe that many physicians will buy it, and it will doubtless be the means of preventing much suffering and of porlonging many lives.

